# Effects of micelle silymarin in corn-soybean meal-based diet on laying hens' performance, egg quality, and blood profile, with comparative assessment of blood absorption rates between powdered and micelle silymarin

**DOI:** 10.1016/j.psj.2024.104029

**Published:** 2024-06-26

**Authors:** Golam Sagir Ahammad, Se Yeon Jang, In Ho Kim

**Affiliations:** Department of Animal Resource and Science, Dankook University, Cheonan, Choongnam 330-714, South Korea

**Keywords:** absorption rate, blood profile, egg quality, laying hen, micelle silymarin

## Abstract

Micelle silymarin (**MS**) is recognized for its diverse range of beneficial properties, which encompass anti-inflammatory, antioxidant, hepatoprotective, and antidiabetic effects. The main objective of this study was to examine the effects of micelle silymarin on the performance, egg quality, blood profile, and absorption rate of silymarin in laying hens. In experiment 1: 288 Hy-Line brown laying hens, 28 wk old, were utilized for this experiment. The hens were randomly allocated into 3 dietary treatment groups, with each group comprising eight replicates of 12 hens, each housed in individual pens with access to feed and water. Over a 12-wk feeding trial, the hens were provided with a basal diet supplemented with different levels of MS: 0, 0.03, and 0.06%. In experiment 2: For this experiment, 192 Hy-Line Brown laying hens were divided into 2 dietary treatment groups, with each group comprising eight replications of 12 hens. The dietary treatments were: TRT1, basal diet + powder silymarin 4%; TRT2, basal diet + MS 4%. From the first experiment, the findings revealed that incorporating micelle silymarin (**MS**) into the hens' diet significantly increased egg weight at wk 6 (*P* < 0.05). Similarly, at wk 12 and throughout the entire experiment, significant effects were observed on downgraded egg count, egg production, egg weight, and feed conversion ratio (**FCR**) (*P* < 0.05). Moreover, Haugh Units (**HU**) and albumen height showed a linear improvement (*P* < 0.05) at wk 4 with MS supplementation. Furthermore, there was a linear increase in egg yolk color, albumen height, and eggshell thickness at wk 8 with MS supplementation (*P* < 0.05). Furthermore, a layers-fed diet supplemented with MS showed a linear increase (*P* < 0.05) in HU, egg weight, yolk color, albumen height, eggshell strength, and eggshell thickness in wk 12. Regarding blood profile parameters, the study revealed linear reductions for aspartate aminotransferase (**AST**), alanine aminotransferase (**ALT**), and lactate dehydrogenase (**LDH**) (*P* < 0.05), whereas there was a tendency for albumin, triglyceride, and cholesterol (*P* < 0.10). In the second experiment, it was observed that the blood absorption rate of silymarin was higher in TRT2 compared to TRT1 at 2- and 4-h intervals following administration. In summary, increasing MS supplementation in the diet of laying hens enhanced egg production, egg quality, and blood profile. Additionally, silymarin absorption was higher in its micelle form than in its powder form.

## INTRODUCTION

The use of growth-promoting antibiotics in poultry production has been associated with potential benefits for production performance (Rafiq et al., 2022). However, concerns have arisen regarding their adverse effects on both birds and humans (Phillips et al., 2004). Consequently, many countries have implemented strict bans on these antibiotics. In response, researchers are actively seeking alternatives that are beneficial for birds and ultimately for humans. In this context, Milk thistle (*Silybum marianum*) emerges as a promising alternative due to its abundance of bioactive compounds (Bijak, 2017). Among these compounds, silymarin, a flavonoid complex extracted from *Silybum marianum* seeds, has gained attention for its potential health benefits in humans and animals (Ranjan and Gautam, 2023). The use of botanical extracts as dietary supplements in animal nutrition has gained popularity due to their reported positive impacts on growth performance, physiological functions, and the well-being of animals (Hashemi and Davoodi, 2011).

Silybin, sourced from the plant *Silybum marianum* of the Asteraceae family, has shown efficacy in addressing a range of liver ailments and acts as an antioxidant by neutralizing free radicals and preventing lipid peroxidation (Gillessen and Schmidt, 2020). Research has shown that silybin reduces the formation of free radicals in injured cells, which may explain its hepatoprotective properties (Vargas-Mendoza et al., 2014). Previous research has suggested that dietary supplementation with botanical extracts rich in bioactive compounds can positively influence growth parameters, egg quality characteristics, and physiological responses in poultry (Darmawan et al., 2022). Phytogenic feed additives containing flavonoids have been shown to serve multifaceted roles, including acting as immune system regulators, antimicrobials, antimutagens, antioxidants, and growth promoters (Biswas et al., 2024). For instance, Gholamalian et al. (2022) demonstrated that adding 30 g/kg of MS to the diet led to a notable enhancement in growth performance, egg quality, egg storage capacity, and blood parameters. Moreover, Faryadi et al. (2021) showed that supplementation of the diet with 200 mg/kg of MS improved hen performance and egg quality. Research indicates that MS seed extract can modulate physiological processes and enhance the overall health of laying hens (Wang et al., 2020). To date, no comparative studies have assessed the absorption rates of different forms of silymarin in the blood of laying hens. Traditionally, the poultry industry has used powder-type silymarin in feed formulations. However, research has shown that silymarin has poor water solubility (Lee et al., 2017). In response, researchers are exploring different silymarin formulations to enhance its water solubility for use in poultry (Di Costanzo and Angelico, 2019). Garg et al. (2022) noted that the micelle‐type silymarin is higher soluble than hydrophobic silymarin. Therefore, we hypothesize that including MS in the diets of laying hens may improve growth performance, egg quality, blood profile, and absorption rate.

This study aims to investigate the effects of dietary supplementation with MS in a corn-soybean meal-based diet on the performance, egg quality, and blood profile of laying hens (Experiment 1). Furthermore, we conducted a comparison of the absorption rates of powdered and micelle silymarin in poultry (Experiment 2)

## MATERIALS AND METHODS

The research conducted in this study adhered to the approved research protocol (DK-1-2316) as per the guidelines of the Animal Care and Use Committee at Dankook University in Cheonan, Republic of Korea.

### Experiment 1

#### Preparation of MS

The MS supplement used in this study was sourced from the Synergen company located in Gyeonggi-do, Korea. It consisted of 10.8% silybin, 16.3% silydianin, and 7.0% silychristin, with a pivotal content of 250 g/Kg. The processing methods involved the following steps:

1. Milk thistle seeds were pressed, and the resulting powder underwent infiltration with alkaline water.
2. The milk thistle powder, after infiltration with alkaline water, was extracted using acetone. The extract was then filtered and concentrated to obtain a concentrated liquid.
3. A nonpolar solvent was utilized to extract the concentrate. Upon separation, both a nonpolar solvent layer and an acetone layer were obtained. The acetone layer was then concentrated and dried to yield silymarin, with the product containing 80% silymarin.
4. The silymarin was subsequently micellized using variable emulsifiers. The resulting product contained a minimum of 20% micelle-type silymarin and a minimum of 2% silymarin.

#### Birds, Husbandry, and Experimental Diets

In this study, 288 laying hens of the Hy-Line brown hybrid, all 28 wk in age, were randomly assigned into three different dietary treatment groups, with each group consisting of eight replicates of 12 hens. This trial lasted for 12 wk. Housing for the hens consisted of individual pens, each sized 38 cm in width, 50 cm in length, and 40 cm in height. The temperature was regulated at 26°C, with a lighting regime consisting of 16 h of light and 8 h of darkness. Nipple drinkers and removable trough feeders were installed in each pen, enabling the hens to freely access both feed and water. The experimental basal diet used for the experiment was prepared in mash form and was formulated in accordance with National Research Council standards for laying hens (NRC, 1994). The ingredients and calculated analysis of the basal diet are presented in Table 1. Experimental diets consisted of 1) CON (basal diet); 2) TRT1: CON + 0.03% MS; and 3) TRT2: CON + 0.06% MS.

**Table 1:** Composition of laying hen diets (as fed-basis).

Item	Basal diet
Ingredients (%)	
Corn	61.39
Soybean meal	16.39
Corn gluten meal	4.94
Wheat bran	3.60
Tallow	0.97
Monodicalcium Phosphate	1.65
Limestone	9.75
Salt	0.25
Lysine (80%)	0.30
Methionine (50%)	0.26
Vitamin mix^1^	0.20
Mineral mix^2^	0.20
Choline (50%)	0.10
Total	100.00
Calculated value	
Metabolic energy, kcal/kg	2800
Crude protein, %	16.50
Crude fat, %	3.43
Crude fiber, %	2.89
Crude ash, %	4.57
Calcium, %	4.08
Phosphorus, %	0.68
Available phosphorus, %	0.45
Lysine, %	0.88
Methionine, %	0.45
Methionine+Cystine, %	0.79

1 Provided per kg of diet: vitamin A, 8,000 IU; vitamin D3, 3,300 IU; vitamin E, 20 mg; vitamin K3, 2.5 mg; vitamin B1, 2.5 mg; vitamin B2, 5.5 mg; vitamin B6, 4 mg; vitamin B12, 23 mg; biotin, 75 mg; folic acid, 0.9 mg; niacin, 30 mg; D-calcium pantothenate, 8 mg

2 Provided per kg of diet: Fe, 40 mg as ferrous sulfate; Cu, 8 mg as copper sulfate; Mn, 90 mg as manganese oxide; Zn, 80 mg as zinc oxide; 1.2 mg as potassium iodide; and Se, 0.22 mg as sodium selenite

#### Sampling and Laboratory Analysis

The experiment involved the recording of daily feed intake and the total number of eggs laid, including any downgraded eggs (those with defects or abnormalities) for each replication. Egg production percentage, FCR, and egg weight were calculated daily based on the recorded data for each replicate. Egg mass was calculated by the following formula:

Eggmass=eggproduction(%)×meaneggweight(g)

Egg quality assessments were conducted at the end of the 4th, 8th, and 12th wk during the experimental period. To evaluate egg quality, 30 eggs from each treatment, free from shell defects, and cracks, were selected at 17:00 h. Egg quality measurements were performed on the same day at 20:00 h.

An eggshell color fan was employed to determine eggshell color, and visual assessments were conducted by various expert members. Haugh Units, eggshell strength, and yolk color were measured using a digital egg TESTER, specifically the DET6500. Eggshell thickness was measured using a dial pipe gauge manufactured by Ozaki MFG. Co., Ltd., Tokyo, Japan.

Blood samples were collected from a total of 48 hens, with 16 hens per treatment group (2 hens per replicate), using a wing vein puncher at the initial stage (1 wk) and the final stage (12 wk) of the trial. These samples were placed in K2-EDTA heparinized tubes (BD Vacutainer, Plymouth, Devon, UK). Following collection, the samples were centrifuged at 3,000 *g* for 15 min to isolate the plasma, which was subsequently transferred into Eppendorf tubes and preserved at -20°C until subsequent analysis. The levels of AST, ALT, alkaline phosphatase (**ALP**), LDH, albumin, triglyceride, and cholesterol were determined using a Konelab 20 analyzer (Thermo Fisher Scientific, Vantaa, Finland), employing commercial diagnostic kits and following the manufacturer's guidelines.

### Experiment 2

#### Birds, Husbandry, and Experimental Diets

In this study, 192 laying hens of the Hy-Line brown hybrid, all 28 wk in age, were randomly assigned into 2 different dietary treatment groups, with each group consisting of eight replicates of twelve hens. Experimental diets consisted of TRT1: basal diet + 4% powder silymarin and TRT2: basal diet + 4% MS. Housing for the hens consisted of individual pens, each sized 38 cm in width, 50 cm in length, and 40 cm in height. The temperature was maintained at 26°C, and the lighting schedule comprised 16 hours of light followed by 8 h of darkness. Nipple drinkers and removable trough feeders were installed in each pen, enabling the hens to freely access both feed and water. The experimental basal diet used for the experiment was prepared in mash form and was formulated by National Research Council standards for laying hens (NRC, 1994). The ingredients and calculated analysis of the basal diet are presented in Table 1.

#### Sampling and Measurement

Blood samples were collected using 5 ml K3EDTA vacuum tubes (Becton Dickinson Vacutainer Systems) from the vein on the brachial wing of each bird at the beginning of the test, 2 h, and 4 h into the experiment. After collection, each sample received the addition of 6 mL of methanol, followed by centrifugation at 10,000 *g* for 10 min at 4°C. After centrifugation, the supernatant was carefully transferred to a new 15 mL tube and subjected to evaporation using nitrogen concentration on a 50°C water bath. Subsequently, the sample was aliquoted with 6 mL of 0.2 M phosphate buffer solution (pH 7.2). Silymarin flavonolignans (CAS No. 65666-07-1) and Naringenin (internal standard, IS) (CAS No. 67604-48-2) were procured from Sigma-Aldrich. Acetonitrile (HPLC grade) and distilled water were obtained from Tedia, while HPLC-grade ethyl acetate was purchased from J. T. Baker. Formic acid was sourced from Sigma-Aldrich.

A stock solution of silymarin was prepared in acetonitrile at a concentration of 2 mg/mL. Working solutions of silymarin were prepared by adding acetonitrile drop by drop to the stock solution of silymarin to achieve concentrations of 10, 20, 50, 100, 200, 500, 1,000, and 2,500 ng/mL. Naringenin was dissolved in acetonitrile to obtain a 20 ng/mL solution. All stock and working solutions were stored at -20°C during analysis.

Standard calibrations were generated by adding silymarin working solutions to blank plasma, producing final concentrations of 1, 2, 5, 10, 20, 50, 100, and 250 ng/mL. Calibration standards or plasma samples (200 μL) were mixed with 20 μL of IS solution (20 ng/mL naringenin in acetonitrile) and 1 mL of ethyl acetate. The combination was vigorously vortexed for 5 min before being centrifuged at 16,000 *g* for another 5 min. The organic portion was moved to a fresh tube and desiccated using a SpeedVac (Christ RVC 2-25 CDplus; Martin Christ). The residues were reconstituted using 100 μl of mobile phase before injection into a liquid chromatography-mass spectrometry (LC-MS/MS) system.

Silymarin concentration in laying hen plasma samples was determined using an Agilent 6470 triple quadrupole LC-MS/MS system. Chromatographic separation of silymarin from the baseline in laying hen plasma was achieved using a Synergi polar RP column (2.0 mm × 150 mm, pore size: 4 µm; Phenomenex) with isocratic elution of mobile phase consisting of water and acetonitrile (15:85, v/v) with 0.1% formic acid. The column was kept at a constant temperature of 30°C, while the flow rate of the mobile phase was adjusted to 0.2 mL/min. LC-MS/MS analysis was performed using an electrospray ionization (**ESI**) source in negative ion mode with multiple reaction monitoring (**MRM**) transitions at m/z 481.2 → 151.0 for silymarin.

#### Statistical Analysis

All the data collected from Experiment 1 were statistically analyzed in a randomized block design using the generalized linear model's procedure of SAS (SAS Institute). The replicated pen was considered as the unit for this study. Orthogonal polynomials comparisons showed the linear and quadratic effects of increasing dietary concentrations of supplemental silymarin. All data were analyzed with Duncan's multiple range test to check the significance. However, a t-test was conducted in Experiment 2. The variability of data was represented using the standard error of the mean. As a result, *p* < 0.05 was considered as statistical significance, whereas *p* < 0.10 was considered as a trend.

## RESULTS

### Experiment 1

At wk 6, the inclusion of MS in the corn soybean meal-based diet led to a linear an increase in egg weight (*p* < 0.05). Furthermore, at wk 12 and throughout the entire experiment, the addition of MS in the diet resulted in a linear reduction in downgraded egg count, FCR and increases in egg production, and egg weight (*p* < 0.05) (Table 2). Moreover, HU, and albumen height showed a significant tendency (*P* < 0.10) at wk 4 with MS supplementation. Furthermore, there was a tendency for increased egg yolk color and albumen height (*P* < 0.10), as well as a linear increase in eggshell thickness at wk 8 with MS supplementation (*P* < 0.05). Layers fed diet supplemented with MS also showed a linear increase in HU, yolk color, albumen height, eggshell Strength, and eggshell thickness in wk 12 in Table 3 (*P* < 0.05). Regarding blood profile parameters, the study revealed a tendency for reduced AST at the beginning of the experiment. By the end of the study period, there was a significant linear decrease in AST, ALT, and LDH (*P* < 0.05), alongside observed tendencies in albumin, triglycerides, and cholesterol (*P* < 0.01), as detailed in Table 4

**Table 2:** The effect of micelle type Silymarin supplementation on production performance in laying hens.^1^

Items	CON	TRT1	TRT2	SEM^2^	*P* - value^3^	
					Linear	Quadratic
Week 0-6						
Downgraded egg, %	3.3	3.15	2.7	1.2	0.8062	0.9691
Egg production, %	77.4	78.0	78.8	1.9	0.6958	0.8258
Feed Intake, g	109.15	109.26	109.34	1.3	0.7854	0.6734
Egg weight, g	63.2^b^	64.3^ab^	64.8^a^	0.5	0.0308	0.6537
Egg mass, g/h/d	48.91	46.99	48.64	0.8	0.8712	0.7645
FCR	2.19	2.18	2.16	0.05	0.7375	0.8624
Week 7-12						
Downgraded egg, %	3.9^a^	3.4^b^	3.2^b^	1.2	0.0269	0.5049
Egg production, %	73.2^b^	75.3^ab^	76.0^a^	2.3	0.0346	0.3829
Feed Intake, g	109.22	109.45	109.48	1.5	0.7845	0.9765
Egg weight, g	64.2^b^	65.3^ab^	65.8^a^	0.5	0.0308	0.6537
Egg mass, g/h/d	46.99	48.34	48.79	0.7	0.5464	0.8933
FCR	2.32^a^	2.23^ab^	2.19^b^	0.03	<.0001	0.1174
Overall						
Downgraded egg, %	3.6^a^	3.3^b^	2.9^b^	0.2	0.0043	0.1563
Egg production, %	75.3^b^	76.6^ab^	77.4^a^	1.8	0.0069	0.7230
Feed Intake, g	109.18	109.35	109.41	1.1	0.6532	0.9274
Egg weight, g	64.6^b^	68.3^a^	68.8^a^	0.5	<.0001	0.0056
Egg mass, g/h/d	48.64	52.31	53.25	0.6	0.4219	0.5563
FCR	2.25^a^	2.21^ab^	2.18^b^	0.06	0.0023	0.5198

1 Abbreviations: CON, Basal diet; TRT3, CON + micelle type silymarin 0.03%; TRT4, CON + micelle type silymarin 0.06%; FCR, feed conversion ratio.

2 Standard error of means.

a,b Means in the same row with different superscripts differ significantly (*P* < 0.05).

**Table 3:** The effect of micelle type Silymarin supplementation on egg quality in laying hens.^1^

Items	CON	TRT1	TRT2	SEM^2^	*P* - value^3^	
					Linear	Quadratic
Week 4						
Egg Shell color	11.2	11.3	11.0	0.4	0.6827	0.6132
HU	83.4	85.5	86.1	1.2	0.0987	0.6019
Yolk color	7.7	8.1	7.9	0.3	0.5158	0.3492
Albumen height, mm	9.5	9.8	10.4	0.3	0.0556	0.6689
Eggshell Strength, kg/cm^2^	4.55	4.66	4.70	0.23	0.6528	0.9174
Eggshell Thickness, mm^−2^	39.6	40.3	41.0	1.0	0.2210	0.9773
Week 8						
Egg Shell color	10.8	10.9	11.1	0.6	0.6724	0.9610
HU	79.6^b^	83.4^ab^	85.0^a^	1.5	0.0117	0.5337
Yolk color	8.1	8.2	8.7	0.3	0.0954	0.5593
Albumen height, mm	8.9	9.3	9.7	0.3	0.0744	1.0000
Eggshell Strength, kg/cm^2^	4.31	4.36	4.40	0.09	0.4403	0.9601
Eggshell Thickness, mm^−2^	37.8^b^	39.1^ab^	40.5^a^	0.9	0.0290	0.9751
Wk 12						
Eggshell color	11.3	10.9	11.1	0.5	0.7995	0.6601
HU	76.2^b^	83.7^a^	84.1^a^	1.3	<.0001	0.0262
Yolk color	8.3	8.8	9.1	0.3	0.0711	0.8326
Albumen height, mm	6.9^b^	7.6^a^	8.0^a^	0.3	0.0400	0.7267
Eggshell Strength, kg/cm^2^	4.07^b^	4.42^a^	4.44^a^	0.11	0.0199	0.2184
Eggshell Thickness, mm^−2^	35.7^b^	38.6^a^	39.0^a^	0.5	<.0001	0.0448

1 Abbreviations: CON, Basal diet; TRT3, CON + micelle type silymarin 0.03%; TRT4, CON + micelle type silymarin 0.06%.

2 Standard error of means.

a,b Means in the same row with different superscripts differ significantly (P<0.05).

**Table 4:** The effect of micelle type Silymarin supplementation on blood biochemical parameters in laying hens.^1^

Items	CON	TRT1	TRT2	SEM^2^	*P* - value^3^	
					Linear	Quadratic
Initial (week 1)						
AST, U/L	229	235	247	7	0.0748	0.7427
ALT, U/L	2.38	1.75	2.13	0.40	0.6638	0.3225
ALP, U/L	381	344	422	27	0.2888	0.1016
LDH, U/L	1224	1218	1431	121	0.2474	0.4721
Albumin, g/dL	2.15	2.46	2.33	0.16	0.4491	0.2669
Triglyceride, mg/dL	1308	1276	1230	49	0.2788	0.9129
Cholesterol, mg/dL	119	149	130	16	0.6190	0.2322
Finish (week 12)						
AST, U/L	301^a^	279^ab^	255^b^	13	0.0205	0.9399
ALT, U/L	4.13^a^	3.25^ab^	3.00^b^	0.32	0.0277	0.4440
ALP, U/L	468	441	432	42	0.5499	0.8631
LDH, U/L	1901^a^	1673^b^	1484^b^	73	0.0012	0.8328
Albumin, g/dL	2.14	2.48	2.51	0.13	0.0531	0.3457
Triglyceride, mg/dL	1639	1382	1371	96	0.0682	0.3142
Cholesterol, mg/dL	172	157	149	8	0.0787	0.7000

1 Abbreviations: CON, Basal diet; TRT3, CON + micelle type silymarin 0.03%; TRT4, CON + micelle type silymarin 0.06%; AST, aspartate aminotransferase; ALT, alanine aminotransferase; ALP, alkaline phosphatase; LDH, lactate dehydrogenase

2 Standard error of means.

a,b Means in the same row with different superscripts differ significantly (P<0.05).

### Experiment 2

The rate of absorption of silymarin in laying hens is shown in Table 5. Results showed that micelle‐type silymarin showed a higher (*p* < 0.05) absorption rate compared to the powdered-type silymarin in 2nd, 4th h.

**Table 5:** The effect of dietary Silymarin supplementation on absorption rate in laying hens.^1^

Items	TRT1	TRT2	SEM^2^	P-value^3^
Silymarin				
0H	0.00	0.00	-	-
2H	34.25^b^	78.44^a^	1.38	0.0322
4H	16.12^b^	43.96^a^	1.21	0.0483

1 Abbreviations: TRT1, Basal diet + powder silymarin 4%; TRT2, Basal diet + micelle type silymarin 4%; H, hour

2 Standard error of means.

a,b Means in the same row with different superscripts differ (*P* < 0.05).

## DISCUSSION

In this study, MS supplementation in a corn-soybean meal-based diet had a significant impact on the production performance of laying hens. Our study findings are consistent with previous research, as supplementing the basal diet with 0.5g of commercial silymarin per kg led to a notable reduction in FCR, and enhancement in egg production (Hamed et al., 2016). Moreover, Erişir et al. (2016) reported that incorporating 10 g/kg of silymarin seeds into the diet significantly enhanced egg production and decreased FCR in laying hens. Furthermore, Hashemi Jabali et al. (2018) observed a significant reduction in feed conversion and improvement in egg production in leghorn-type crossbreeds (initiating at 40 wk) when administering 30 g of milk thistle flour per kilogram of feed during the 70-d experiment. Micelle silymarin (**MS**) likely played a role in reducing the incidence of downgraded eggs by enhancing eggshell thickness and strength in our study. The observed increases in egg production rates and egg weight suggest that MS supplementation improves nutrient utilization and metabolic efficiency in laying hens (Wang et al., 2020). MS supplementation in laying hen diets has the potential to optimize nutrient utilization, enhance metabolic efficiency, improve egg quality, and reduce stress, potentially resulting in a lower FCR. Conversely, Quarantelli et al. (2009) stated that supplementations with 0.2 and 0.4 g/kg of Silymarin had no significant effect on the growth performance of laying hens. Similarly, Talib Abdulwahid et al. (2021) noted that 0.5% of silymarin/kg feed had no significant effect on the productivity of laying hens. Moreover, Šťastník et al. (2019) observed a significant increase in egg production with the supplementation of 7% milk thistle in the laying diet, but no effect on FCR was reported. Furthermore, Ahammad et al. (2024) reported that supplementation with 0.06% MS had no significant effect on production performance in laying hens. The varying results could be attributed to several factors, including differences diet composition, type of silymarin, variations in dosage, study duration, and genetic variation among the hens.

The quality of eggs is a crucial factor in the poultry sector, influencing the effectiveness, output, and general well-being of hens. In our study, MS supplementation had a significant effect on HU, yolk color, albumin height, eggshell thickness, and eggshell strength. Haugh Unit (**HU**) is widely accepted as a measure of albumen quality, with higher values indicating fresher and higher-quality eggs (Li et al., 2017; Özek et al., 2011). Diets rich in protein, vitamins, and minerals can contribute to the production of eggs with higher HU values (Kopacz et al., 2021). Oxidative stress can contribute to protein degradation in the egg white, leading to decreased albumen viscosity and lower HU values (Obianwuna et al., 2022). By scavenging free radicals and reducing oxidative damage, MS may help preserve the structural integrity of egg proteins, thereby maintaining higher HU values indicative of fresher eggs (Surai, 2015). The observed enhancement in HU values due to silymarin supplementation suggests a positive influence on egg albumen quality (de Menezes et al., 2012). It was also supported by our study showing that albumen height increased significantly by MS supplementation in the diet. This aligns with the findings of Gholamalian et al. (2022) who observed improvements in eggshell strength and thickness, as well as HU, with the inclusion of 30 g/kg of milk thistle meal in the diet. Moreover, Šťastník et al. (2019) reported significant improvements in albumin height and HU values with the supplementation of 7% milk thistle in the diet. The modulation of yolk color depends on carotenoid deposition or metabolism in the egg yolk (Dansou et al., 2023). Carotenoids undergo enzymatic conversions, such as cleavage and oxidation, within the hen's body before deposition in the egg yolk (Surai et al., 2001). Silymarin may promote the accumulation of specific carotenoids or their derivatives in the yolk while also preventing oxidative damage to the pigments responsible for yolk color due to its antioxidant attributes (Taleb et al., 2018). The uterus plays an integral role in the formation and maturation of the eggshell, influencing its ultimate structure and quality (Hashemi Jabali et al., 2018). In this regard, Mirbod et al. (2017) proposed that the remarkable implications of plant-extracted bioactive substances on protein metabolism and holistic antioxidant strength could contribute to the health of the oviduct or uterus, leading to evident enhancements in eggshell quality. Silymarin's hepatoprotective properties can contribute to improved calcium metabolism and utilization in laying hens, thereby enhancing mineralization of the eggshell (Vargas-Mendoza et al., 2014). Moreover, silymarin supplementation has been shown to alter the composition and activity of the gut microbiota (Jahanian et al., 2021). A balanced and diverse gut microbiota is essential for optimal nutrient absorption and overall gut health (Valdes et al., 2018). Thus, by enhancing the absorption of essential minerals like calcium and improving the health of the oviduct or uterus, MS supplementation may contribute to improved eggshell strength and eggshell thickness.

Monitoring AST, ALT, and LDH levels in laying hens is important for assessing liver and muscle health, nutrient metabolism, and overall bird welfare. Elevated levels of AST and ALT in the blood can indicate liver damage or disease. However, in the present study, we observed a tendency for changes in albumin, triglyceride, and cholesterol levels, with a significant reduction of AST, ALT, and LDH at the end of the experiment. This aligns with previous research findings by Hamed et al. (2016), where supplementation of a basal diet with 0.5g of commercial silymarin per kg diet significantly affected ALT, cholesterol, and triglyceride levels in laying hens. Additionally, Tedesco et al. (2004) reported notable decreases in AST, ALT, and ALP activities in broilers exposed to an aflatoxin-contaminated diet upon supplementation with silymarin. Furthermore, adding 0.5% and 1% milk thistle seeds to broiler chicken diets led to a significant reduction in AST activity in the blood fluid of the experimental versus the control broilers (Amiri Dumari et al., 2014) . Moreover, Hashemi Jabali et al. (2018) observed that the inclusion of 60 g/kg of milk thistle meal in the diet resulted in a decrease in serum cholesterol levels and a reduction in plasma HDL cholesterol levels in laying hens. Likewise, after the inclusion of 0.06% silymarin in the ration, Shanmugam et al. (2022) noted a significant decrease in blood cholesterol levels in broilers by d 35. Correspondingly, the inclusion of 30 g/kg of milk thistle (*Silybum marianum*) meal (**MTM**) in the diets resulted in a significant reduction in serum cholesterol levels, as observed by Gholamalian et al. (2022). Conversely, conflicting results were reported by Šťastník et al. (2019), who found that supplementation of 7% milk thistle in the ration had no significant impact on the hematological parameters of laying hens. On the other hand, including 1,000 mg/kg of silymarin in the feed, as conducted by (Blevins et al., 2010), showed no influence on the blood profile of laying hens. Moreover, supplementing the diet with 0.10% MS did not result in any significant changes in the blood profile of weaning pigs (Dang et al., 2022). The varying results can be attributed to differences in diet compositions and forms of silymarin used in the study. In this study, MS supplementation significantly reduced AST, ALT, and LDH levels, indicating its ability to protect the liver from damage caused by toxins and oxidative stress (Gillessen and Schmidt, 2020). By preserving liver function, silymarin may indirectly impact AST, ALT, and cholesterol levels. A healthier liver is better equipped to regulate cholesterol metabolism and enzyme production, such as AST and ALT (Vargas-Mendoza et al., 2014), which is aligned with the current study. Moreover, research suggests that silymarin may inhibit the activity of the enzyme HMG-CoA reductase (Li et al., 2019), which is crucial for cholesterol synthesis in the liver. By reducing the production of cholesterol in the liver, silymarin can lead to lower levels of cholesterol in the bloodstream. The observed increase in albumin levels suggests enhanced protein synthesis and improved overall metabolic health in the hens (Gholamalian et al., 2022). Similarly, the tendencies observed in triglyceride and cholesterol levels indicate potential improvements in lipid metabolism (Wang et al., 2020). Despite their lack of statistical significance, these changes suggest a potential positive impact of MS supplementation, warranting further investigation.

Our study showed that the absorption rate of micelle-type silymarin in blood was higher than that of powder silymarin. Silymarin constituents, such as silybin, silychristin, and silydianin, have limited solubility in water, which can hinder their absorption from the gastrointestinal tract (Sornsuvit et al., 2018). Micelle-type formulations encapsulate silymarin within surfactant molecules, forming micelles that solubilize the hydrophobic constituents, thereby increasing their availability for absorption (Garg et al., 2022). Micelle-type formulations typically have smaller particle sizes and higher surface areas compared to powdered formulations (Bagheri et al., 2018). The enhanced dispersion and dissolution of silymarin in gastrointestinal fluids in micelle-type formulations promote efficient interaction with absorptive surfaces of the intestinal epithelium. This interaction is facilitated by surfactant molecules interacting with cell membrane lipids, thus aiding in the transport of silymarin constituents (Di Costanzo and Angelico, 2019). This facilitated transport mechanism, mediated by micelle formation, promotes efficient absorption of silymarin into the bloodstream, leading to higher systemic availability. It was found that a higher absorption rate in the blood was found in micelle‐type silymarin compared to powdered silymarin after the 1st, 2nd, 4th, 8th, 12th, and 24th h of feeding in finishing pigs (Hossain et al., 2023). Additionally, the absorption rate in blood was very high when micelle-type silymarin was administered in dogs (Yu et al., 2010). The exact mode of action of micelles-silymarin in poultry remains unclear. Nonetheless, the findings from our study are preliminary and necessitate validation through further research.

## CONCLUSIONS

Incorporating 0.06% micelle-type silymarin (MS) into a corn-soybean meal-based diet for 12 wk significantly improved production performance and egg quality, positively influencing AST, ALT, LDH levels, and showing trends in albumin, triglyceride, and cholesterol levels. Diets containing 0.03% and 0.06% MS also resulted in higher egg characteristics such as weight, HU, albumen height, eggshell thickness, and strength compared to controls, suggesting potential use of these MS levels in the poultry industry for laying hens. In addition, the incorporation of MS demonstrated higher blood absorption rates compared to powder-type silymarin. Further research is essential to fully elucidate the absorption mechanisms of different silymarin formulations in laying hens.

## DISCLOSURES

The authors declare no conflicts of interest.
